# Testimonial injustice in the treatment of children’s testimonies: inconsiderate and unjust cases

**DOI:** 10.1007/s42048-025-00226-4

**Published:** 2025-07-11

**Authors:** Peter Paul Elicor, Bettina Bussmann

**Affiliations:** 1 Kindergarten am See, Salzburg, Austria; 2 Department: Philosophie GW, Universität Salzburg, Salzburg, Austria

**Keywords:** Testimonial injustice, Children, Inconsiderate treatment, Unjust treatment, Silencing

## Abstract

In this article, we propose a framework for understanding testimonial injustice as it applies to children. Building on the work of Fricker and other scholars in the field, we maintain that age-based discrimination in testimonial exchanges undermines children as knowers, disadvantaging them from being heard and recognized as credible sources of knowledge. To contribute to this discourse, we distinguish *inconsiderate* and *unjust* responses to children’s testimonies to refine our evaluative categories in protecting children from epistemic harm while also maintaining conceptual clarity. We define inconsiderate treatments as dismissals or disbelief rooted in misguided personal assumptions, conflicting options for action, or unreflected norm conflicts rather than systemic age-based prejudice. Unjust treatments, by contrast, involve similar dismissiveness but are driven by personal bias reinforced by systemic discrimination. Although this distinction also applies to adults, our framework highlights the unique vulnerability of children, whose developmental stage and social status make them particularly susceptible to unintentional disregard and structural injustice. We conclude with a discussion of interpersonal and structural silencing to illustrate how testimonial injustice is committed against children.

## Introduction

The concept of epistemic injustice has generated discussions about the extent to which its fundamental features can be extended and applied to testimonial exchanges involving children *qua* knowers. Existing literature shows that epistemic injustice arises not only from negative stereotypes related to race and gender but also from age. Drawing from Miranda Fricker, it is generally agreed that children suffer distinctly as knowers when age-based discrimination creates asymmetrical testimonial interactions, placing them at a disadvantage in terms of being listened to and communicating knowledge. Thus, it is argued that children, too, suffer testimonial injustice in situations where their testimonies are accorded a lesser degree of credibility than they deserve.

Fricker describes testimonial injustice as something that occurs when a person gives a “deflated level of credibility” to another due to identity prejudice (2007, p. 1). This type of epistemic injustice occurs when a speaker is prejudged based on a stereotype inconsistent with the available evidence and irrelevant to their epistemic capacities. Like social or class discrimination, identity prejudice targets an individual’s membership in a social group, causing others to dismiss what they know and communicate, even if it is true. While a credibility deficit due to negative stereotypes often happens incidentally, testimonial injustice captures a particular kind of credibility deficit that systematically follows a person (Fricker 2007, p. 27). In other words, epistemic injustice is caused by identity-based discrimination that recurs in various social contexts, not only in one-off testimonial exchanges.

That children suffer from epistemic injustice, particularly testimonial injustice, is a view held by several scholars who have made much headway in this field. The existing literature supports the argument that epistemic injustice applies not only to gender and race but also to age. Except for a few, most research in this area follows Fricker’s definition of epistemic injustice, i.e., “a wrong done to someone specifically in their capacity as a knower” (2007, p. 1). In the case of children, epistemic injustice happens when they are accorded low epistemic credibility due to a stereotype based on their age. Below is an overview of the main points on epistemic injustice vis-à-vis children in different contexts, including education, healthcare, culture and politics, digital environments, and law.

In educational contexts, children suffer testimonial injustice when they are not correctly heard (Murris 2013), when hearers fail to give epistemic attention to their contributions, leading to “epistemic attention deficit” (Smith and Archer 2020), when teachers systematically grant them less epistemic credit due to negative identity prejudices (Kotzee 2017), when their capacity as knowers affects their personal development and self-formation, resulting in ‘formative epistemic injustice’ (Nikolaidis 2021), or when they are denied “hopeful epistemic trust,” a kind of trust given to a child that helps develop their epistemic agency (Brick 2020).

In the context of health and medicine, epistemic injustice is done to children when their accounts of themselves are discounted in clinical settings because they are unreliable, volatile, or unstable (Harcourt 2021), when they are pressured to conform to an identity category and deprived of the means to understand and communicate their own social experience due to the epistemic authority of medicine (Klyve 2019), or when their testimonies are dismissed based on the assumption that they are poor givers of testimony (Carel and Györffy 2014).

In culture and politics, children experience epistemic injustice when they are excluded from political activities, such as voting (Baumtrog 2021), or prevented from participating in political decision-making (Martin 2018). This exclusion is based on the common assumption that children lack the maturity and competence to engage in political matters, as they are perceived not to have passed the threshold of maturation and development.

Research on epistemic injustice in digital environments has not explicitly addressed children’s unique experiences. However, these observations likely apply particularly to adolescents exposed to such environments. In this context, testimonial injustice occurs when children’s accounts of themselves are discredited in favor of a more ‘objective’ and quantified representation of their biography (Origgi and Ciranna 2017), or when they struggle to manage their online identities, making it difficult for them to maintain hermeneutical control over how they are represented online (ibid.).

Finally, in the context of law and forensics, children face epistemic injustice when they are subject to identity prejudice, such as the assumption that their testimonies are inherently untrustworthy despite little supporting evidence (Baumtrog and Peach 2019), or when they are treated as deficient testifiers due to the belief that they are overly suggestible and therefore unreliable witnesses (Burroughs and Tollefsen 2016).

The above-mentioned claims are based on the premise that a child, as a knower, suffers testimonial injustice when a hearer assigns them low epistemic credibility due to a widespread prejudice about their age. Just like in cases involving women and people of color, epistemic injustice against children is an instance of a credibility deficit. However, not everyone agrees with this line of argument. Bartlett, for example, thinks that “conceiving of testimonial injustice purely as a credibility deficit obscures the key dynamics of testimonial injustice as it relates to children” (2021, p. 4). For him, there are occasions when a hearer is justified in assigning a child low epistemic credibility. We will elaborate on this point in the third section of the paper. For now, we maintain that a purely credibility-based approach to testimonial injustice in children’s experiences is problematic.

To this end, we distinguish *inconsiderate* and *unjust* responses to children’s testimonies to refine our evaluative categories in protecting children from epistemic harm while also maintaining conceptual clarity concerning the nature of testimonial injustice. We define inconsiderate treatment as the dismissal, disregard, or disbelief of children’s testimonies based on misguided personal assumptions, conflicting options for action, or unreflected norm conflicts rather than on systemic age-based prejudice. Unjust treatment, on the other hand, refers to similar dismissive responses that are grounded in personal bias and reinforced by systemic age-based prejudice. Underlying this distinction is the fact that children have unique vulnerability in testimonial contexts where their developmental stage and social status intersect, increasing their susceptibility to unintentional disregard and systemic epistemic injustice. While this framework is explicitly tailored to children, many elements may also apply to adults. However, the emphasis on developmental factors and power dynamics underscores how children are more acutely affected, even though similar influences can sometimes impact adults.

## Some preliminary considerations

Children’s epistemic and communicative abilities depend highly on their age and level of maturity. Experts working with children’s testimonies in legal settings know that several factors should be considered, such as cognitive development, communicative competence, socio-emotional maturity, memory, and suggestibility (Saywitz 2002). Indeed, this does not discount the fact that some children are more knowledgeable than most adults in other areas. However, in general, a reasonable disbelief in what children may know, even if it leads to a dismissal of their testimonies, does not automatically result in testimonial injustice. When mental health is at stake, Harcourt argues that while listening is essential, there is “more to being listened to than simply believed” (2021, p. 734). Of course, it is important to acknowledge and remedy the various situations in medical settings where children’s descriptions of their condition are not given the serious attention they deserve. These situations are often caused by the widespread belief that children are irrational, easily influenced, possess limited reasoning abilities, have flawed or non-existent memories, and have a restricted capacity for language.

It is also important to note that the cognitive maturity of a child, which is dependent on age, significantly influences their degree of knowledge and understanding, as well as how their testimony is received and assessed. Research on developmental psychology shows that children’s cognitive abilities, including memory, attention span, and ability to understand complex information, develop gradually over time (Daniels and Clarkson 2010). This directly affects how they perceive, remember, and articulate their experiences. Emotional maturity, particularly the ability to regulate emotions, which develops with age, also influences how children convey their experiences (Thompson 1994). Moreover, social understanding, which includes the ability to perceive others’ perspectives and intentions, affects how children interpret interactions and communicate their experiences (Carpendale and Lewis 2006). Another consideration is that children’s communication skills also undergo significant development. Their vocabulary, grammar, and ability to form coherent narratives evolve with age, affecting their capacity to articulate their experiences clearly and comprehensively (Zauche et al. 2016). Because of these age-related developmental factors, children’s testimonies should be evaluated differently than those of adults, necessitating active listening, patient inquiry, and a willingness to believe children, even when their first accounts are not immediately apparent.

Further, the nature and context of the testimonial exchange are critical in determining the credibility of a child’s testimony. The context of the testimonial exchange, whether formal (e.g., courtroom) or informal (e.g., family talk), can significantly impact the child’s comfort and reliability. For instance, children interviewed in a mock courtroom showed increased anxiety and poorer performance on memory tests than those interviewed in a more familiar school setting (Edelstein et al. 2002). Power dynamics are also important, as the hearer’s relative power and authority can influence the credibility attributed to the child. Children may feel frightened when adults hold great authority, affecting their desire and ability to deliver accurate testimony (Saywitz 2002). Thus, the circumstances surrounding a testimonial exchange also make it challenging for children to articulate their experiences in ways easily understood by adults.

When children are involved, developmental factors such as cognitive and linguistic abilities, socio-emotional maturity, memory capacity, and the power dynamics that shape testimonial exchanges with them require closer scrutiny. While these considerations may also be relevant in adult contexts, their impact on children is significantly more pronounced.

## The problem of credibility-based testimonial injustice

Imagine a scenario in which an adult’s testimony is disbelieved and dismissed solely based on a negative stereotype towards their race. This simplified schema illustrates the two primary conditions of testimonial injustice: *credibility deficit* and *identity prejudice*. In this scenario, the knower-speaker experiences epistemic disadvantage because their race, say Asian, has no bearing on their capacity as a knowing agent and their credibility in speaking about something they know. Moreover, the extent of injustice is amplified when the subject is already in a disadvantaged position and has an important stake in the decisions from which they are excluded, as well as suffering other social injustices (Byskov 2021). This example illustrates that when a hearer allows a negative stereotype to cloud his judgment of the speaker’s credibility, resulting in the dismissal of the latter’s claims despite evidence to the contrary, he causes the speaker to suffer testimonial injustice.

In cases involving children, credibility deficit and identity prejudice are insufficient or too restricted to account for children’s epistemic experiences. To illustrate, consider the same scenario, but this time, the knower-speaker is a child, and their testimony is disbelieved and dismissed because of their age. Despite the presence of the two conditions, we would be hard-pressed to determine whether the hearer’s dismissal of the child’s word constitutes testimonial injustice, unlike the scenario involving an Asian adult. The presence of testimonial injustice is difficult to determine if the case is taken at face value, as other important questions must be considered. These include: What does the child know? Is the child’s knowledge reliable and relevant to the testimonial exchange? What is the child’s age and level of maturity? And is the child actively involved in both their testimony and the outcomes of the exchange? It is necessary to consider these questions since disbelieving someone based on age is not inherently wrong. It appeals to common sense that most children, particularly the very young, do not have the same level of cognitive and emotional maturity and social understanding as many adults; and, all other things being equal, most children’s epistemic capacities differ from those of most adults primarily due to developmental factors associated with age.^1^

Consider another scenario: During a class, a 10-year-old student shares a view that the teacher finds irrelevant to the subject at hand, which he then dismisses as one of those childish views commonly asked by children of their age. Has the teacher committed testimonial injustice? This is a case of an age-based prejudicial credibility deficit. However, claiming that cases like these constitute testimonial injustice is excessive. If credibility deficit and identity prejudice are taken as the sole conditions for testimonial injustice, then the teacher is culpable, albeit undeservedly. On this view, the teacher’s internal act of discounting the student’s testimony, motivated by a prejudicial perception of the question as irrelevant or ‘childish,’ would constitute an instance of epistemic injustice. However, this interpretation is misleading. Instead, we argue that the teacher’s mental act of dismissing the student’s question, rooted in a perception of its triviality rather than identity-based prejudice, does not fulfill the criteria for testimonial injustice. While the response may be pedagogically problematic, it does not rise to the level of epistemic harm envisioned by Fricker’s account of testimonial injustice. To conclude otherwise would risk overstretching the concept, conflating the internal attribution of credibility with the external speech act of dismissing a child’s contribution. Hence, a purely credibility-based testimonial injustice applied to children’s testimonial experiences is problematic. On this note, Bartlett argues that epistemic harm does not occur in assigning children low epistemic credibility because of prejudice but rather in failing to “actively inquire into their assertions” (2021, p. 2).

Departing from a credibility-based account of testimonial injustice, the following section examines three categories that characterize testimonial exchanges involving children: (1) cases where dismissing their words is neither inconsiderate nor unjust, (2) cases where it is inconsiderate, and (3) cases where it is unjust. Drawing these distinctions is crucial when evaluating testimonial exchanges involving children because the consequences can be profound: failing to make these distinctions risks either downplaying genuine testimonial injustices or overstretching the concept in ways that render it philosophically unhelpful. Labeling every dismissive response as unjust blurs the boundary between moral wrongdoing and developmental misunderstanding or conflicting options for action, undermining the precision needed to identify actual cases of testimonial injustice. At the same time, excusing all dismissals as merely inconsiderate risks ignoring the real harms children may face when their knowledge is routinely underestimated.

## Various treatments of children’s testimony

Returning to the scenario above, a child’s testimony is disbelieved and dismissed because of their age. In this scenario, additional crucial information is required concerning the speaker and hearer of the testimonial exchange. Thus, we recall the questions posed above, which are underpinned by implicit criteria such as relevance, reliability, and involvement.^2^ As previously mentioned, in the context of children’s testimony, these conditions take on heightened significance due to developmental factors (e.g., cognitive and linguistic abilities, socio-emotional maturity, and memory) and the pervasive age-based prejudices (e.g., ageism, deficit assumptions towards childhood), which significantly influence a child’s ability to communicate and be heard, differing from the factors that typically affect adults (e.g., gender and race).

Equally important are the conditions surrounding the hearer and their response. What is the reason for the dismissal? What is the hearer’s relation to the child? Does it stem from systemic age-based prejudice, a deeply ingrained bias towards young people, or merely a momentary lapse of judgment influenced by contextual factors such as stress, misunderstanding, or misguided assumptions? These questions highlight key conditions for determining whether a hearer has committed a testimonial injustice, namely, the reasons for the dismissal, the hearer’s relationship to the child, and the hearer’s testimonial actions. These need to be carefully examined and contextualized because testimonial injustice is an act committed by a hearer in testimonial exchanges. While some of these questions are also applicable in cases involving adults, they highlight the unique challenges children face in testimonial exchanges. Aside from developmental differences between a child speaker and an adult hearer, children are often subjected to power dynamics and frequently in positions where adults hold more authority. Also, due to their vulnerability, children may be more likely be influenced by authority figures and less likely to have their testimonies taken seriously in challenging environments.

### Neither inconsiderate nor unjust treatments

In some situations, children’s statements are dismissed, doubted, or minimized but done reasonably and responsibly rather than rude or unfair. In these cases, the dismissal or doubt typically arises from a pragmatic or cautious approach rather than an intent to harm or disrespect the child. While the child’s testimony may be downplayed or not fully trusted, the adult does not act out of malice or bias but instead acts in the child’s best interest, especially when the child is very young. Unlike most adults, children are still undergoing crucial developmental processes, which must be carefully considered when evaluating and responding to their testimonies. As mentioned, their epistemic, cognitive, and communicative abilities largely depend on their age and maturity level.

One of the most common examples is dismissing a child’s statement due to its perceived lack of relevance to the testimonial context or the child’s limited involvement in it. In these contexts, the child’s assertions carry no weight in the testimonial exchange they seek to contribute to, particularly when they are not directly involved. In such cases, disregarding their knowledge is a pragmatic decision, primarily when clarity and accuracy are necessitated. The child is not being mistreated; instead, the dismissal reflects a reasonable and fair evaluation of what they know, with no apparent epistemic or moral harm to the child or others.

Imagine a group of adults discussing some events in the Paris Olympics. A 5-year-old child, overhearing the conversation, interjects with a comment about their favorite game board. While the child’s statement may be accurate, it is nevertheless irrelevant to the adults’ discussion. Additionally, the child is not involved in the topic or its implications. Dismissing their statement in this context, but with a gentle redirection back to their play, would neither constitute an inconsiderate nor unjust act. Similarly, in a family context, when a parent dismisses a child’s statement because they do not seem to understand what is being talked about thoroughly, and there is evidence to believe so, it would be imprudent to think that the parent has discriminated against the child.

Moreover, treatments of children’s testimony that are neither inconsiderate nor unjust may involve dismissals based on reasonable caution rather than unfair treatment of the child. These dismissals typically stem from a belief that the child’s cognitive, emotional, or experiential stage means they may not have the full understanding of the situation. Adults in these situations may withhold complete trust in the child’s account, not because they reject entirely or unfairly dismiss the child’s assertions but because they are taking a cautious approach that includes carefully understanding the context. When dismissals are based on reasonable caution, they are typically based on a thoughtful evaluation and prudent response to a testimonial exchange and not dismissive in a harmful way. Adults in these situations are not undermining the child’s credibility; instead, they are simply exercising carefulness to ensure the child’s well-being.

Consider another example: In a neighborhood setting, a 10-year-old child claims they did not start a conflict with another child, instead blaming the other for provoking the incident. Rather than immediately accepting this account, the parent responds thoughtfully, emphasizing the need to speak with both children and any witnesses before reaching a conclusion. This cautious approach reflects an understanding that children often have limited or biased perspectives in conflict situations. By gathering all sides of the story, the parent ensures fairness and avoids misinterpretation, thoughtfully addressing the situation with care and consideration for its complexity.

When adults respond to children’s testimony with reasonable caution, which includes seeking additional context, considering developmental factors, and carefully weighing the relevance of a child’s perspective, they are engaging in a fair and responsible evaluation of what the child is communicating. These responses reflect an effort to treat the child with respect while recognizing the complexities of interpreting testimony. In such cases, dismissing a child’s statement is not a denial of their voice but a thoughtful judgment about how that voice fits into the broader context.

### Inconsiderate treatments

Inconsiderate treatments occur when a child’s testimony is ignored or dismissed due to misguided assumptions about the child rather than explicit or systemic age-based prejudice. These assumptions include beliefs that the child is too emotional, too naïve, or ‘simply ignorant about what is going on,’ even when their input is meaningful and potentially helpful. In such cases, adults may not intend to be dismissive or biased, but their actions still result in the child’s voice being overlooked. People often face time constraints, routines, and situations where they must follow certain norms. In these circumstances, they may choose to overlook a child’s assertions to comply with expectations or obligations. For example, a teacher under time pressure or preoccupied with other responsibilities might unintentionally neglect a student’s concern. Many such instances show that inconsiderate treatment is not always intentional. The key distinction here is that the dismissal stems from a lack of thoughtful engagement with the child’s perspective rather than from a deliberate or deeply ingrained belief that children are categorically less credible. This makes the treatment inconsiderate as it reflects a failure to acknowledge the value of the child’s contribution.

Consider this example: A group of children playing in a park witness a crime. When the police arrive, one child, eager to help, tries to describe what they saw. However, the officer focuses their questioning on adults who arrived later and may not have observed the event as clearly. The child, despite possessing crucial information, is also heard. However, their testimony is eventually dismissed, preventing the transmission of relevant and reliable information and potentially obstructing justice for the victim and allowing the perpetrator to go free. It is an inconsiderate treatment if the reason for dismissal is based on an honest but mistaken belief, i.e., the officer wrongly assumes the child misunderstood the situation. Even if we assume that the police officer’s belief is motivated by a temporary bias, this alone does not automatically constitute testimonial injustice. Another condition must also be established: the nature of the relationship between the child and the hearer. Since the police officer holds a position of authority, it is important to ask whether this power dynamic exerted undue influence on the officer’s evaluation of the child’s testimony. If it did, then it may contribute to a testimonial injustice; if not, the dismissal may be considered inconsiderate.

Children are often seen as unreliable witnesses due to stereotypes about their suggestibility, trustworthiness, and competence. In many societies, children’s social identity is shaped by ageist stereotypes that influence how adults, especially those possessing a specific authority over them, assess their credibility. Generational hierarchies and traditional norms further reinforce children’s epistemic inferiority, prioritizing the authority of elders over children’s perspectives. Because of these widespread age-based prejudices, children’s testimonies require greater attention and care to ensure their accounts are considered fairly rather than dismissed outright.

Consider another example: A child tries to tell their parent about a distressing experience they had with an adult caretaker. The child struggles to articulate what happened, but they are clearly upset and seeking their parent’s care and support. However, the parent, who would typically pay attention to their child, is preoccupied with work and dismisses the child’s concerns, assuming they are exaggerating or misinterpreting a minor incident. This response constitutes an inconsiderate treatment of the child, as it overlooks the emotional urgency and vulnerability expressed in their attempt to communicate. The child is not only a knower of their own experience but also directly involved in the event they are trying to report. By brushing off the child’s account, the parent fails to acknowledge the child’s epistemic agency and emotional need for validation and support.

However, it would not constitute unjust treatment if the dismissal stems from an honest but mistaken belief; for example, if the parent genuinely, though incorrectly, assumes that the child is simply being ‘too emotional.’ The parent’s dismissal can be understood as a momentary lapse of judgment rather than an instance of deliberate or systemic injustice. In this case, the parent is momentarily distracted (e.g., overwhelmed by work or other stressors) and fails to fully register the emotional weight of their child’s attempt to communicate. While the response may be inconsiderate, it is not rooted in prejudice or a pattern of disbelief but rather in a temporary failure to respond appropriately due to competing demands on the parent’s attention.

Unlike adult testimonies, children’s testimonies require distinct evaluative approaches due to their developing cognitive and linguistic abilities, as well as their emotional and social understanding, which may hinder their attempts to articulate their experiences in a way that is easily comprehensible to adults, even those closest to them, such as parents and guardians. To truly understand a child, adults must recognize not only the intended message or underlying meaning behind their words but also their emotional and practical needs, which may not always be expressed clearly. This is especially important when dismissing a child’s knowledge stems from the adult’s misunderstanding or inaccuracies in interpreting the child’s testimony.

### Unjust treatments

Unjust treatments are those that undermine a child’s credibility or agency without proper justification. It is unjust to dismiss children’s knowledge, especially if relevant to the testimonial exchange; the child is directly or indirectly involved in the exchange and its outcomes, and the hearer’s dismissal is based on a personal bias and reinforced by systemic age-based prejudice. In such cases, the child is wrongfully denied recognition as a credible informant, not because of the content of their testimony but because of biased assumptions tied to their age. Unjust treatments of children’s testimonies are often rooted in societal structures or norms that devalue children’s testimonies due to their age rather than being a momentary lapse of the hearer’s judgment. The severity of this injustice is further magnified if the hearer holds a position of authority over the child.

Moreover, unjust treatments against children’s testimonies adversely affect their developing epistemic abilities and compromise their integrity as whole persons, diminishing their chances of developing in ways that maximize their full potential. Not only do they affect children’s evolving identities (e.g., age, cognitive abilities, and social roles) and flexible identities (e.g., religion, gender, and cultural identity), but also their enduring identities (e.g., race, sex, distinct physical features). The impact of testimonial injustice on children is multifaceted, affecting their identities both in the present and long term. They can lead to lasting developmental consequences when done systematically over time and with increasing severity.

The following section explores interpersonal and institutional child-silencing as forms of testimonial injustice. However, we first briefly present two cases where this concept applies.

Since 1998, two men have sexually abused 32 children at a campsite in Lügde, North Rhine-Westphalia, without being apprehended. It was not until 2019, nearly two decades later, that they were caught and put behind bars. The investigation discovered that the youth welfare officers supposed to ensure the children’s safety and well-being “looked far too little” and “listened far too little” to them.^3^ Despite the reports from the victims themselves and concerned individuals, neither the police nor the responsible agencies had taken any proactive measures to ensure that the victims’ reports were properly investigated.

In an analogous case, an American Catholic priest who had ministered in the diocese of Naval, Biliran, Philippines, for 37 years was arrested in 2018 for supposedly sexually abusing dozens of boys. His alleged crimes “were known” in the town, “but no one would take action, talk to the victims, or try to stop the abuse for years.”^4^ Much like the previous case, the local church and state authorities responsible for addressing the issue and preventing further damage failed to act despite previous reports. There are numerous other examples of child maltreatment that have stayed unresolved for years, even decades, due to either cover-up or inaction.^5^ Abusing children is one wrongdoing, a direct harm that subjects a child to physical, emotional, sexual, or psychological harm by the abuser. However, ignoring or dismissing their account is another. It is indirect but equally grave, denying child protection and justice and also enabling abusers to continue unchecked.

Children, particularly the very young, are often unable to recognize and comprehend abuse, much less speak about it. Often, victims are abused by people who are supposed to be caring for them (Smith and Freyd 2014). In these cases, coming to terms with the abuse and accepting that it happened can be a long process. Some children, particularly those whose parents are prone to incorrectly believing them to be lying, would choose to remain silent to avoid punishment (Goodman-Brown et al. 2003). Those who chose to speak out were met with disbelief, and their concerns were dismissed or downplayed. For this reason, many child victims believe disclosing their experience is not worth the potential risks, which include feelings of shame, guilt, and anxiety from reliving the pain during the process of speaking about it.^6^ In a study, children who disclosed abuse asserted that “their suffering does not end with telling” (Berliner and Conte 1995, p. 383). However, because many forms of child abuse occur discreetly and leave little to no visible evidence, a child’s disclosure is often the only way for the abuse to be identified and stopped. Additionally, emotional recovery can only begin when the abuse is reported. Thus, when child victims speak up but are not heard, they are denied both the epistemic power to assert the truth and the agency to take control of their own lives.

## Interpersonal child-silencing

In its broadest sense, silencing occurs when one is barred from communicating. This occurs, for instance, when a person is physically restrained from speaking or when one is denied access to an intended hearer. In less overt ways, silencing is also done by discrediting someone’s word or completely ignoring them. Not all forms of silencing are immoral; however, when silencing is used to prevent a person from speaking an inconvenient truth, like abuse, it becomes an unjust act.

Silencing a victim, especially a child, does not always come in obvious ways. Oftentimes, it would involve subtle manipulations to force the victim to keep the abuse hidden. Moreover, the greater the cost of exposure, the more pressing the need to silence the victim. Migdow observes that “if the perpetrator successfully imbues the victim with shame, then the perpetrator no longer needs to fear exposure. The victim herself will fear exposure” (1994, p. 180). As a result, the child would refuse to speak up because, without their knowledge, they have been silenced. In these cases, silencing violates one’s epistemic capacities, impairing one’s capacity to function as a giver and receiver of knowledge. Emerick claims that “silencing is sometimes a form of epistemic injustice that can result in the violation of the integrity of the person who is silenced by diminishing their epistemic capacities” (2019, p. 29). If silencing is repeated over time, it could “short-circuit their ability to interpret the world in a way that is meaningful and fits with their experience,” which has long-term cumulative and developmental consequences (2019, p. 35). This will gradually impair their ability to develop a healthy sense of self and trust. Thus, prolonged exposure to silencing contributes to the deterioration of an individual’s hermeneutic capacity for interpreting and communicating their experiences.

Interpersonal cases of child silencing are the most basic and widespread, which include imbuing the child with shame, attributing blame or betrayal, making threats, or appealing to the child’s pity, all of which occur on an individual level, either intentionally or unintentionally, actively or passively. Underlying these actions is the pervasive notion that adults are superior to children, giving them control over their ability to speak, be heard and believed. This justifies treating children as not credible to be treated as givers of knowledge, which is inherently prejudicial against their epistemic agency. In societies where children are treated inferior, such prejudice inevitably undermines their ability to influence their lives and the world around them, which, in effect, normalizes child silencing. According to van der Kolk, “being prevented from articulating what they observe and experience, traumatized children will organize their behavior around keeping the secret, deal with their helplessness with compliance or defiance, and acclimate in any way they can to entrapment in abusive or neglectful situations” (2005, p. 404). Thus, for child victims, silencing does not only undermine their exercise of epistemic agency and autonomy but, most importantly, affects their efforts toward psychological and emotional recovery. Most child victims deal with trust issues and lack of self-confidence, which inevitably impact the way they form relationships with other people.^7^ Thus, the degree of epistemic harm tremendously impacts their lives, fracturing their social, psychological, and moral-epistemic integrity, inevitably impacting their life prospects.

## Institutional child-silencing

Interpersonal cases of child silencing, are to a large extent, influenced and informed by a society’s collective beliefs and attitudes toward children in general. Some of these seep into the structures of institutions tasked with supervising children’s welfare. Crucially, these unjust treatments of children’s testimony are more insidious when preconditioned by corrupt institutional culture. Certain epistemically corrosive practices, such as cover-ups, are present in institutions (e.g., schools, churches, courts of law, social services, foster homes, and industries like sports and film) to the extent that they are diffused and infused within the social fabric (Smith and Freyd 2014). For this reason, child silencing is also a systemic issue that routinely and pervasively ‘tracks’ children across society, placing them at a disadvantage in terms of being listened to seriously. It is also important to note that such a disadvantage is intersectional, thereby affecting certain children whose other identity markers (e.g., intellectual capacity, gender, race, and language) are socially discriminated against (Baumtrog and Peach 2019).

When a child experiences institutional testimonial injustice, the epistemic harm is not so much tied to whether the institution gave their testimony low credibility but to whether their testimony was actively listened to and inquired about regardless of their age and manner of disclosure. It is a fact that children’s testimonies are often deemed unbelievable especially when it involves persons with high institutional positions (Smith and Freyd 2014). In such cases, the act of silencing the child seeks to harm, not as a primary motive, but as a means to achieve another goal, that is, to shield the institution. One could argue that in these instances, silencing is committed out of an implicit recognition that a child’s claim may be true and that, due to the high cost of exposure, it must be silenced to protect both the individual in question and the institution’s reputation. What this reveals is that testimonial injustice against children, when taken to the institutional level, would be too narrow if conceived purely as credibility-based.

Institutional child silencing constitutes explicit mechanisms intended to suppress a child’s word actively. For instance, interventions such as settling complaints accompanied by a nondisclosure agreement or gag clauses effectively prevent victims from speaking about their experiences (Ozor 2022). Although this practice is legally permissible in many countries, it hinders the victim’s emotional recovery by further deepening their silence. Equally important is that it also blinds the public to the reality of such abuses. Moreover, child silencing also happens as a result of inaction or non-intervention, whereby assertions and complaints made by children are ignored and not given the necessary attention.^8^ In this passive silencing, child disclosures are not reported to the appropriate authorities, resulting in unnecessary delays in initiating active intervention. This practice stems from various institutional factors, such as the absence of policies or precedents, insufficient competent personnel, a lack of training or clear instructions, or overall incompetence within the institution. While tackling testimonial injustice on the interpersonal level is fundamental, addressing this problem on the institutional level is more important and, to some extent, more effective in introducing changes to the existing culture that is epistemically unfavorable to children.

## Conclusion

In the foregoing, we clarify what qualifies as injustice in the context of children’s testimony to distinguish between morally significant cases and those that are not, thereby avoiding the risk of overstretching the concept of testimonial injustice. Our distinction between inconsiderate and unjust cases also addresses the theoretical and practical problems that arise from applying a purely credibility-based account of testimonial injustice to children’s testimonial experiences. We hope that the framework presented in this paper will help facilitate decision-making about what does and does not count as an injustice.

It cannot be emphasized enough that testimonial injustice extends beyond situations of abuse, encompassing various settings where children’s voices are unfairly disregarded due to systemic age-based prejudice. This makes it important to recognize the importance of listening to and believing children in all aspects of their lives. To create an environment that truly listens to and values children’s voices, adults and institutions must adopt a framework of ethical engagement that prioritizes children’s epistemic agency and well-being. This requires moving beyond simply acknowledging children’s voices to actively dismantling the systemic and interpersonal structures that often silence them.

For adults, specially trained professionals like educators, this involves approaching every interaction with a child as a potential learning opportunity. This means actively listening, asking clarifying questions, and avoiding dismissive language or assumptions about a child’s understanding. Adults must be particularly attentive when a child discloses abuse or harm, recognizing the immense courage and vulnerability involved. In such cases, immediate action, including reporting to relevant authorities and providing emotional support, is crucial. Thus, creating safe spaces where children feel empowered in sharing their thoughts and feelings without fear of judgment or reprisal is important. This can be achieved by promoting open, honest communication within families and communities. In these environments, children learn about their rights, how to recognize and respond to unsafe situations, and the importance of respectful communication.

Meanwhile, institutions, as powerful agents of socialization, bear a significant responsibility in creating environments that protect and empower children. Identifying, critically assessing, and effectively reforming unjust social institutions are important lest we risk playing “corrective whack-a-mole,” that is, addressing interpersonal instances of testimonial injustice while letting unfair institutional practices remain unchecked (Carel and Kidd 2021, p. 474). This requires examining and reforming internal policies and practices that may perpetuate child silencing, such as nondisclosure agreements or inadequate reporting mechanisms. Institutions should prioritize transparency and accountability, ensuring that allegations of abuse or neglect are thoroughly investigated and appropriate action is taken. Therefore, it is crucial to invest in training and resources that equip professionals working with children to effectively engage with children’s testimonies. This includes education on child development, trauma-informed care, and strategies for conducting age-appropriate interviews.

Involving children in matters that affect them can be done by providing platforms for their voices in policy discussions, community initiatives, and other relevant spaces. By recognizing children as active agents of change, institutions can foster a sense of agency and empowerment, enabling them to contribute to building a more just and equitable world. Ultimately, dismantling the structures that perpetuate testimonial injustice against children requires a collective societal shift toward recognizing their experiences and valuing their unique perspectives.

## Footnotes

1 Vygotsky, for example, stresses the importance of social interaction and cultural context in children’s cognitive development. Children’s thinking differs from adults’ because of their continual development and the influence of their social environment. They acquire and develop epistemic abilities in a social setting and with the guidance of more knowledgeable others (The Zone of Proximal Development). See Vygotsky (1978).

2 Relevance, reliability, and involvement are conditions present in all accounts of testimonial injustice. The cases that occasion testimonial injustice involve accounts that are not merely random, unreliable, and irrelevant testimonies from uninvolved parties. Instead, the speakers in these cases not only possess relevant and reliable knowledge of which they are involved, but they also have a stake in the outcomes of the testimonial exchange. Fricker’s examples illustrate this well: Tom Robinson in *To Kill a Mockingbird* is unjustly convicted because the jury dismisses his crucial testimony about his alleged involvement in a crime. Similarly, in *The Talented Mr. Ripley*, Marge Sherwood’s account of her partner’s whereabouts is disregarded despite her direct knowledge of the situation (Fricker 2007, pp. 23, 86).

3 Lukas Eberle, *DER SPIEGEL*, A. G. February 11, 2022. (S+) “Fall Lügde: So Haben Die Behörden Im Missbrauchsskandal Versagt. Fall Lügde: So haben die Behörden im Missbrauchsskandal versagt.” https://www.spiegel.de/panorama/gesellschaft/fall-luegde-so-haben-die-behoerden-im-missbrauchsskandal-versagt-a-5fc99122-f5b8-4d2f-83f2-4c9df6601cfd.

4 After serving the local church for 37 years, he died while on trial in January 2022. Cullen, S. December 21, 2021. *Clerics Get Away With Child Abuse In the Philippines—UCA News. *ucanews.com. https://www.ucanews.com/news/clerics-get-away-with-child-abuse-in-the-philippines/95428. See also Petilla, D. February 13, 2022. “Requiem for an Indicted Priest” *Inquirer News*. https://newsinfo.inquirer.net/1553917/requiem-for-an-indicted-priest?fbclid=IwAR3_oj4aalccPc-aMxY46LedR3mo0n7jNTEfHbcMWLO_ZA4m7uNI8LUmd7I.

5 Consider, for example, the decade-long suppression of evidence of child sexual abuse involving 1400 children in Rotherham. See Stevenson, Luke. August 26, 2014. https://www.communitycare.co.uk/2014/08/26/child-sexual-exploitation-suppressed-ignored-rotherham-finds-damning-inquiry/. Another case is the abuse of children in the care of Lambeth Council (south of London), where children were maltreated from the 1960s to the 1990s. Many staff members who were supposed to take care of the children “failed to act when they knew about sexual abuse and showed little compassion for the victims.” See Butler, Patrick, July 27, 2021. https://www.theguardian.com/uk-news/2021/jul/27/hundreds-of-children-abused-while-in-care-of-lambeth-council-inquiry-finds.

6 According to Moon, the average time it takes for someone to come forward and talk about their abuse is 24 years, and some people never come forward at all (Moon 2019).

7 See McCrory et al. (2011), who highlight the fact that childhood maltreatment has been reliably associated with poor outcomes not only in physical and mental health but also, equally important, in social functioning.

8 There are numerous cases whereby complaints were shelved “in a drawer in an executive’s office” instead of being acted upon to ascertain their veracity as prescribed by law. See https://athleteafilm.com/wp-content/uploads/2020/06/ATHLETEA_Guide.pdf.
